# miRNAs as Candidate Biomarker for the Accurate Detection of Atypical Endometrial Hyperplasia/Endometrial Intraepithelial Neoplasia

**DOI:** 10.3389/fonc.2019.00526

**Published:** 2019-06-21

**Authors:** Simona Giglio, Viviana Annibali, Roberto Cirombella, Omar Faruq, Stefano Volinia, Claudia De Vitis, Margherita Pesce, Donatella Caserta, Angela Pettinato, Filippo Fraggetta, Andrea Vecchione

**Affiliations:** ^1^Department of Clinical and Molecular Medicine, “La Sapienza” University, Sant'Andrea Hospital, Rome, Italy; ^2^Department of Neurosciences, Mental Health and Sensory Organs, Centre for Experimental Neurological Therapies (CENTERS), “La Sapienza” University, Sant'Andrea Hospital, Rome, Italy; ^3^Department of Internal Medicine, Biosystems Analysis, LTTA, Department of Morphology, Surgery and Experimental Medicine, Università Degli Studi, Ferrara, Italy; ^4^Department of Medical-Surgical Sciences and Translational Medicine, “La Sapienza” University, Sant'Andrea Hospital, Rome, Italy; ^5^Pathology Unit, Cannizzaro Hospital, Catania, Italy

**Keywords:** microRNAs, endometrial hyperplasia, endometrial cancer, biomarkers, SMAD4, TGF-β pathway

## Abstract

Endometrial cancer is the most common gynecologic malignancy in developed countries. Estrogen-dependent tumors (type I, endometrioid) account for 80% of cases and non-estrogen-dependent (type II, non-endometrioid) account for the rest. Endometrial cancer type I is generally thought to develop via precursor lesions along with the increasing accumulation of molecular genetic alterations. Endometrial hyperplasia with atypia/Endometrial Intraepithelial Neoplasia is the least common type of hyperplasia but it is the type most likely to progress to type I cancer, whereas endometrial hyperplasia without atypia rarely progresses to carcinoma. MicroRNAs are a class of small, non-coding, single-stranded RNAs that negatively regulate gene expression mainly binding to 3′-untranslated region of target mRNAs. In the current study, we identified a microRNAs signature (miR-205, miR-146a, miR-1260b) able to discriminate between atypical and typical endometrial hyperplasia in two independent cohorts of patients. The identification of molecular markers that can distinguish between these two distinct pathological conditions is considered to be highly useful for the clinical management of patients because hyperplasia with an atypical change is associated with a higher risk of developing cancer. We show that the combination of miR-205, −146a, and −1260b has the best predictive power in discriminating these two conditions (>90%). With the aim to find a biological role for these three microRNAs, we focused our attention on a common putative target involved in endometrial carcinogenesis: the oncosuppressor gene SMAD4. We showed that miRs-146a,−205, and−1260b directly target SMAD4 and their enforced expression induced proliferation and migration of Endometrioid Cancer derived cell lines, Hec1a cells. These data suggest that microRNAs-mediated impairment of the TGF-β pathway, due to inhibition of its effector molecule SMAD4, is a relevant molecular alteration in endometrial carcinoma development. Our findings show a potential diagnostic role of this microRNAs signature for the accurate diagnosis of Endometrial hyperplasia with atypia/Endometrial Intraepithelial Neoplasia and improve the understanding of their pivotal role in SMAD4 regulation.

## Introduction

Worldwide, endometrial cancer (EC) represents 4% of all cancers in women and is the most common malignant tumor of the female genital tract in industrialized countries (1). The etiology of EC is not yet fully understood, although there is some evidence that molecular modifications and hormonal influences contribute to its initiation and progression (2).

Endometrial cancer is divided into two major classes: estrogen-dependent tumors (type I, endometrioid endometrial carcinomas) that represent 80% of cases and non-estrogen-dependent (type II, non-endometrioid endometrial carcinomas) that account for the rest. EC type I is thought to develop via precursor lesions along with the increasing accumulation of molecular genetic aberration (3, 4).

Recently, the World Health Organization classification of tumors (WHO) (5) divided endometrial hyperplasias into two categories: hyperplasia without atypia (Benign Hyperplasia, BH) and atypical hyperplasia/endometrioid intraepithelial neoplasia: (AH/EIN) (5).

Indeed, AH/EIN is most likely to progress to type I endometrial carcinoma (~30%), and has been reported to be associated with invasive EC in 62% of endometrial biopsy (6), whereas BH rarely progresses to EC (<5%) (7). Therefore, discerning between these two entities has significant clinical implications (8).

Unfortunately, recognition of atypia in endometrial hyperplasia is subjective among pathologists with a low inter observer reproducibility (<50% in almost all studies) (9, 10).

Although the recent two-tier classification of these entities by WHO (5, 11) improved reproducibility, management of endometrial pre-cancers is compromised by a longstanding debate.

MicroRNAs (miRs) are a class of small, non-coding, single-stranded RNAs that negatively regulate gene expression mainly binding to 3′-untranslated region (UTR) of target mRNAs at the post-transcriptional level (12). Several studies showed that they are important in many biological processes, thus their aberrant expressions are closely associated with the development, invasion, metastasis, and prognosis of various cancers, including EC (13–17).

Up until now, several miR signatures have been documented in either normal or neoplastic endometrium, but the role of miRs in endometrial hyperplasia with or without atypia remains poorly understood (18–22). In the present study, we investigate the hypothesis that changes in miRs may represent useful biomarkers for the diagnosis of AH/EIN.

## Materials and Methods

### Endometrial Tissue Samples and Patients

Eighty-five archived formalin-fixed, paraffin embedded (FFPE) tissue blocks of BH (41 cases), and AH/EIN (44 cases), were obtained from the Pathology Department of Sant'Andrea Hospital and Ospedale Cannizzaro, Catania from 2004 to 2013. Patient's age ranged from 37 to 84 years, with a median of 56 years. This study was authorized by the institutional ethics committee board at S. Andrea Hospital Rome, Italy (Aut. #168/03). Written informed consent was obtained from all patients enrolled.

The selected cases were randomly divided into a training set (23 BH, 19 AH/EIN) and into a validation set (21 BH, 22 AH/EIN).

Hyperplasia was macro or laser-microdissected, were appropriate, for this study.

### RNA Extraction

Total RNA, including miRs fraction, was extracted from FFPE tissues using the High Pure miRNA isolation kit (Roche) according to the manufacturer's instructions. RNAs concentration were assessed using Nanodrop (ThermoScientific).

### Affymetrix Gene Chip miRNA Array

RNA quality and purity were assessed with the use of the RNA 6000 Nano assay on Agilent 2100 Bioanalyzer (Agilent). Briefly, 500 ng of total RNA was labeled using FlashTag Biotin HSR (Genisphere LLC) and hybridized to GeneChip® miRNA 2.0 Arrays. The arrays were stained in the Fluidics Station 450 and then scanned on the GeneChip® Scanner 3000 (Affymetrix, USA).

### Microarray Data Analysis

The statistical analysis was performed by Transcriptome Analysis Console (TAC) software (Thermo Fisher Scientific).

To survey outliers that could disturb the dataset, a Principal Component Analysis (implemented by means of R statistical software) was performed and its visualization, which led to the knowledge of which subjects needed to be excluded from the dataset. MicroRNA probe outliers were defined from the manufacturer's instructions (Affymetrix, USA), and further analysis included data summarization, normalization, and quality control using the web-based miRNA QC Tool software (Affymetrix).

The microarray data has been submitted and assigned a GEO omnibus accession number GSE85105.

### Reverse Transcription and Quantitative Real-Time PCRs

Each sample was reverse-transcribed using miRNA miRCURY LNA Universal RT kit (Exiqon) according to the manufacturer's protocol.

Reverse transcription and quantitative real-time PCRs were performed for miRNAs using miRCURY LNA Universal RT microRNA PCR LNA primers set with miRCURY LNA cDNA Synthesis Kit II and ExiLENT Syber Green master mix, in triplicate (Exiqon). RNU48 (U48) was used to normalize input total small RNA.

Expression of each miR was presented as the ratio between miR and RNU48 (RQ). The relative miRs expression was calculated using the ΔΔ*C*t method. At least three separate experiments were performed, and each sample was assayed in triplicate.

### Cells Transfections

Pre-designed Pre-miR (miR Precursors) for each miR was obtained from Ambion (ThermoFisher Scientific). A negative-control miRNA mimic [Pre-miR miRNA negative #1 (Ambion, ThermoFisher Scientific)] was used to address the specificity of the observed effect to the specific miR sequence. Cells were transfected using Lipofectamine RNAimax (ThermoFisher Scientific).

### Protein Extraction, Western Blotting, and Antibodies

Hec1a cell lines were obtained from ATCC (Atcc, HTB-112) and cultured according to the manufacturer's protocol. Total cell extracts with RIPA buffer (Sigma Aldrich) were collected at 24 h and analyzed by western blot to assess proteins expression levels. Briefly, Hec1a cells were rinsed in ice-cold PBS and subsequently lysed in ice-cold RIPA lysis buffer (Sigma Aldrich) and Complete inhibitor (Roche). Proteins were analyzed on pre-cast polyacrylamide gel (Bio-Rad), transferred onto nitrocellulose membranes (Bio-Rad) and blocked with 5% BSA (Sigma Aldrich), and incubated with specific primary antibodies.

Polyclonal antibody against SMAD4 (sc-7966, Santa Cruz Biotechnology) diluted 1:200. Monoclonal antibody against PAX2 (aJ1589a, Abgent) diluted 1:1,000. Monoclonal antibody against Pten (560002, BD Biosciences) diluted 1:1,000.

To normalize protein loading, membranes were probed for 1 h at room temperature with an anti-vinculin antibody (sc- 25336, Santa Cruz Biotechnology).

Secondary antibodies (labeled HRP anti-rabbit or anti-mouse, Bio-Rad) were incubated for 45 min at room temperature and revealed with chemiluminescent ECL method (Bio-Rad).

Digital images of autoradiography were acquired with ChemiDOC XRS (Bio-Rad).

### Plasmids and Constructs

The 3′UTR of the SMAD4 gene was obtained from GeneArt Gene Synthesis (Invitrogen, Thermo Fisher Scientific) by cloning 900bp of SMAD4 3′UTR into pMir-vector (Promega) giving rise to the pMir-3′UTRSMAD4 construct.

Site-direct mutagenesis into the miR-205, miR146a, and miR-1260b binding sites of the SMAD4 gene 3′UTR were introduced using GeneArt Site-Directed Mutagenesis PLUS System kit (Thermo Fisher Scientific) according to manufacturer's instructions. The primers used were:

FP 5′CTTCACCTGTTATGTAcctgccAATCATTCCAGTGC3′

RP 5′GCACTGGAATGATTggcaggTACATAACAGGTGAAG3′

FP 5′GCTGATTTTAAAGGCAGAGAAccgtcgAAAGTTAATTCACC3′

RP 5′GGTGAATTAACTTTcgacggTTCTCTGCCTTTAAAATCAGC3′

FP 5′GTTATTCCTAGTGacccgtTGTTGATGAAGTATACTTTTCCCC3′

RP 5′GGGGAAAAGTATACTTCATCAACAacgggtCACTAGGAATAAC3

### Luciferase Activity Assays

Hec1a cells were cultured in 12-well-plates and transfected with 500 ng of pMir-3′UTRSMAD4 wt or mutated plasmid or pMir control vector together with 50 ng of β-GAL vector and 50 pmoles of pre-miR-205, pre-miR-146a, pre-miR-1260b, or pre-miR-negative control#1 (Thermo Fisher Scientific). Transfections were carried out using Lipofectamine 2000 and OPTI-MEM as recommended by the manufacturer (Thermo Fisher Scientific). At 48 h after transfection, luciferase activity was measured using the Luciferase Reporter Assay (Promega). Each transfection was repeated twice in triplicate. Transfection efficiency was corrected to β-GAL expression in all cases.

### Cell Proliferation Assay

Cell proliferation was measured using Muse® Count & Viability Assay Kit and Muse® Cell Analyzer as recommended by the manufacturer (Merck Millipore). Cells were transfected with each pre-miR and the pre-miR-negative control#1 into a 35 mm dish as described below and incubated for 72 h. Three independent experiments were performed in duplicate.

### Transwell Migration Assay

The migration ability of Hec1A cells was determined in a Boyden Chamber. Twenty-four hours after transfection, the cells were seeded into 8 μm Transwells (6.5 mm diameter, Corning) at 5 ×104 cells well with serum-free culture medium. Medium containing 10% FBS was added into the lower chamber and served as the chemoattractant. After incubation for 24 h, the cells remaining on the upper surface of the filter were removed by gently wiping with a cotton swab. The cells migrated through the filter were fixed with methanol, stained with MGG quick staining (Bio Optica), and visualized by an inverted fluorescence microscope.

### Statistical Analysis

Data were expressed as the mean ± SD from at least three independent experiments. Statistical analysis between two samples was performed using Student's *t*-test. Statistical comparisons of more than two groups were performed using one-way analysis of variance (ANOVA).

The diagnostic ability of miRs-205, −146a, and −1260b in diagnosing atypical hyperplasia was examined via the area under the corresponding receiver operating characteristic curve (AUC). All statistical analyses were performed using Graph Pad Prism software (GraphPad software). *p* < 0.05 was considered statistically significant.

## Results

### Dysregulated miRs in BH vs. AH/EIN

**Figure 1:**
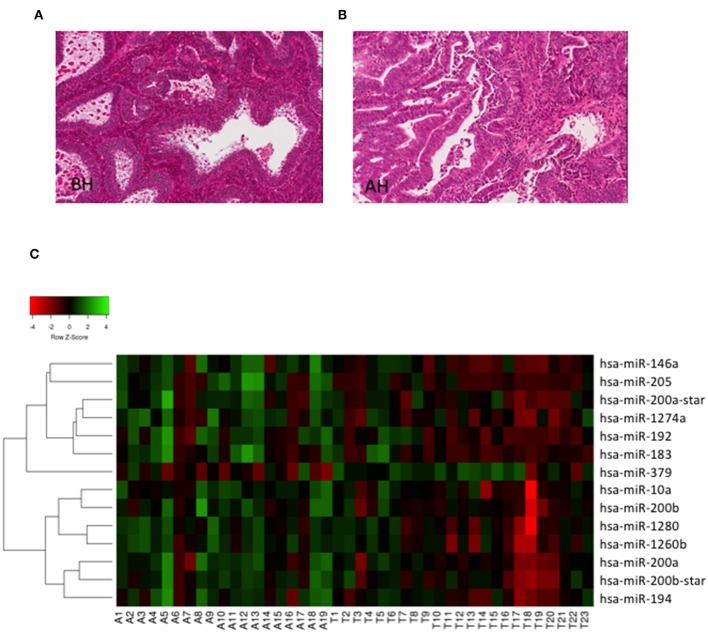
Hierarchical clustering. **(A,B)** Representative H/E stain images of BH and AH (Magnification 20X). **(C)** Heat Map depicting hierarchical cluster analysis of the 14 miRs differentially expressed between AH/EIN (A) and BH (T) identified by microarrays analysis.

To investigate whether miRs could discriminate BH from AH/EIN, we analyzed the expression of 1,105 human miRs (miRbase version 15) in the training set (23 BH, 19 AH/EIN). Examples of BH and AH are presented in Figures 1A,B.

As shown in Figure 1C, we could identify 14 differentially expressed miRs capable of discriminating BH from AH/EIN (FC ≥ 1.5, *p* ≤ 0.05). In particular, 13 miRs were upregulated (miRs-205,-146a, −200b_star, −1274a, −1260b, −200b, −200a, −192, −183, −10, −194, and −200a_star) and 1 (miR-379) was downregulated in AH/EIN compared to BH samples.

Using multiple logistic regression, the statistical significant variables (Age, BMI, Parity, miR values) were assessed in univariate analysis and investigated comparing BH to AH/EIN. No significant correlation was observed (data not shown).

**Figure 2:**
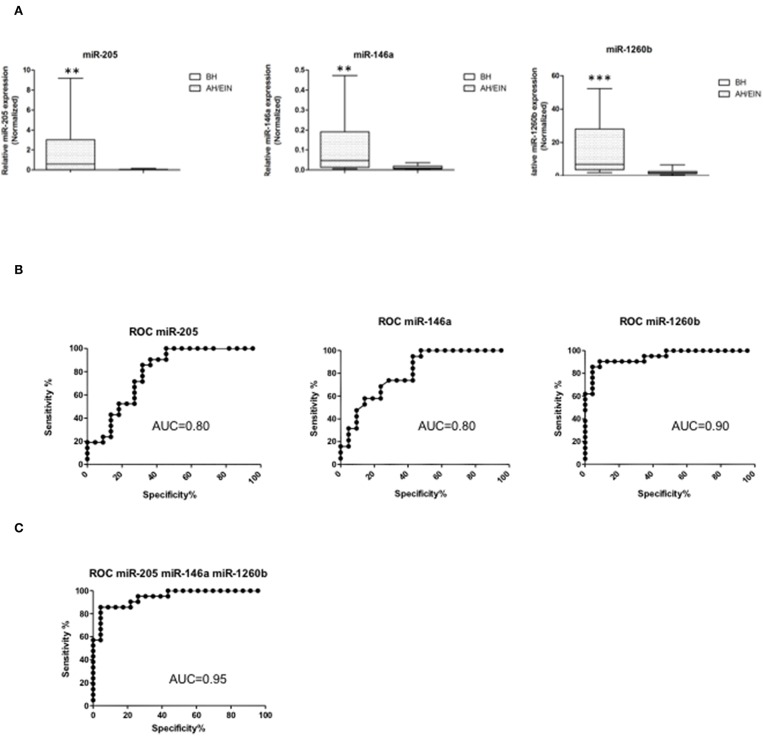
Validation and ROC curve analysis of identified miRs. **(A)** Validated differentially expressed miRs are shown. Expression levels of miRs-205,−146a, and−1260b were significantly higher in AH/EIN compared with BH. The horizontal lines indicate the median value. ^**^p ≤ 0.01, ^***^p ≤ 0.001. **(B,C)** Accuracy for each and for the combination of the three miRs in differentiating AH/EIN from BH, respectively. AUC is shown. All *p* ≤ 0.05.

Differentially expressed miRs were then validated in an independent validation set (21 BH, 22 AH/EIN). Out of the 14 miRs initially identified, we could confirm three miRs all up-regulated (miR-205, −146a, and −1260b) (Figure 2A), suggesting that these miRs could discriminate between the two groups.

To assess the ability of each miR to differentiate between AH/EIN and BH, receiver-operating characteristic curves (ROC) were constructed and the area under the curve (AUC) was calculated. Univariate analysis for each individual miR showed an AUC of 0.8 [95% confidence interval (CI) = 0.66–0.93 *p* = 0.0009] for miR-205, an AUC of 0.8 (95% CI = 0.68–0.94 *p* = 0.0008) for miR-146a, and an AUC of 0.9 (95% CI = 0.88–1.01 *p* < 0.0001) for miR-1260b, respectively (Figure 2B). Performing a multivariate analysis for the combination of the three miRs, we observed an AUC of 0.95 (95% CI 0.88–1.01 *p* < 0.0001) showing that these three miRs have a high predictive power in discriminating AH/EIN from BH (Figure 2C).

### SMAD4 Is a Target of miRs-205, 146a, and 1260b

To investigate a biological role for these miRs in AH/EIN, we searched different prediction algorithms. We found that highly conserved binding sites for each of these miRs were present in the mRNA of the oncosuppressor gene SMAD4, which has been shown to be down-modulated in EC (23).

We used endometrial cancer-derived cell lines Hec1a and tested the endogenous expression of these miRs.

**Figure 3:**
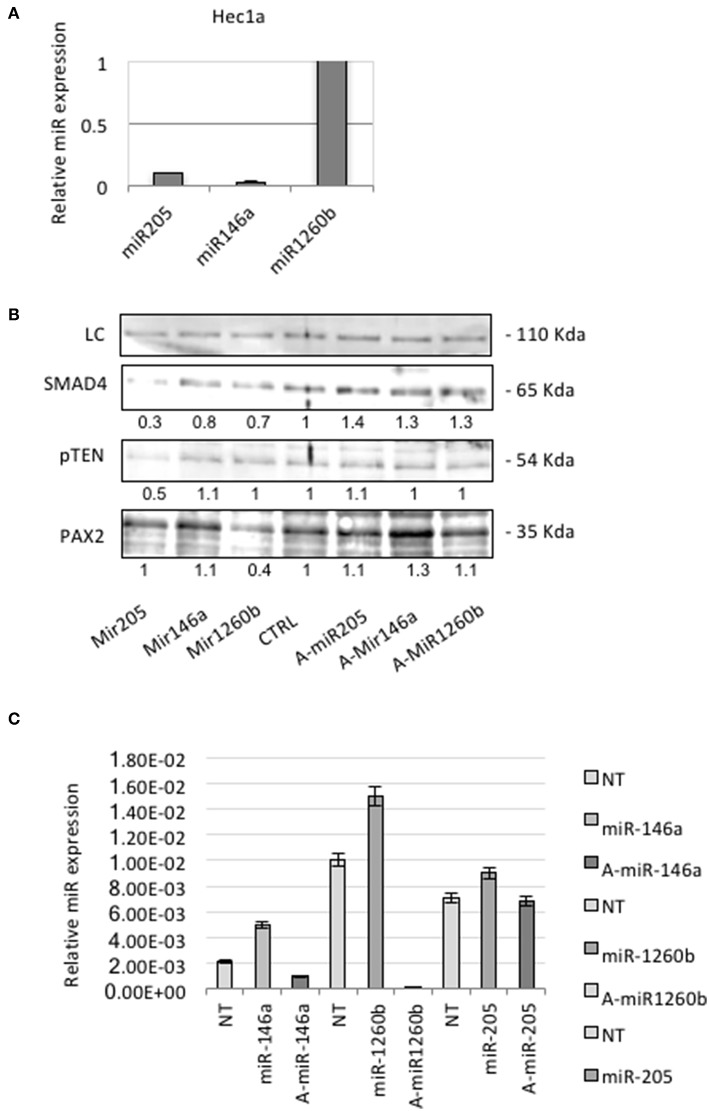
miRs-205,−146a, and−1260b regulate SMAD4 levels. **(A)** Expression levels of each miR were assessed in Hec1a cell line. Relative expression of miRs-205,−146a, and−1260b is reported. Each Ct value is normalized to RNU48. **(B)** Hec1a cells transfected as showed. The Smad4 protein appears as a band at approximately 60 kDa. Actin (~40 kDa) was used as a loading control LC. Densitometry value is reported under each line. **(C)** RT-PCR verification of the transfection efficiency of Hec1a cells transfected in **B**. Each bar shows miR expression normalized to RNU48 ±SD of three independent experiments.

QRT-PCR analysis showed that Hec1a cells expressed detectable amounts of each miRs (Figure 3A). To investigate the effects of these miRs on SMAD4 expression, we transiently transfected pre-miRs-146a, −205, and −1260b or control into Hec1a cells.

In miRs transfected cells, we observed a significant suppression of SMAD4 compared to control (Figure 3B). To further confirm this observation, we transiently transfected Hec1a cells with antagomirs. This resulted in an increase of SMAD4 levels, compared to control (Figures 3B,C), as expected.

Co-loss of both PTEN and PAX2 has been reported in AH/EIN and it has been regarded as the reference markers for its diagnosis (24).

Therefore, we analyzed Pax2 levels by Western blot and we observed a strong protein reduction in miR-1260b transfected cells. There is no evidence of interaction between these two players, but miR binding sites algorithm prediction showed that other Pax family members are the putative target of miR-1260b. We also found that pTEN was downregulated in miR-205 transfected cells confirming previous evidence showing the potential role of miR-205 in regulating PTEN in endometrial tissue (25).

**Figure 4:**
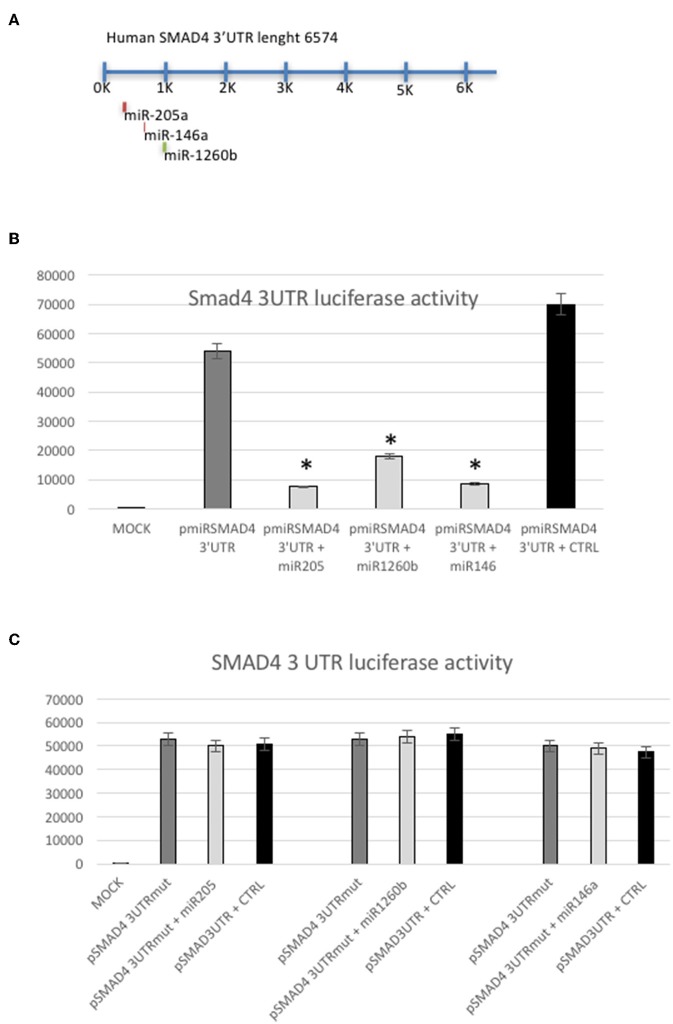
SMAD4 is a target of miRs-205, −146a, and −1260b. **(A)** Schematic representation of predicted miRs binding sites in the SMAD4 3′UTR. **(B)** Luciferase reporter assay in Hec1a cells co-transfected with the reporter gene containing the SMAD4 3′UTR alone (pmiRSMAD4 3′UTR) (gray bar), the SMAD4 3′UTR and miRs-205, −1260b, and −146a (light gray bars), respectively and the negative control (CTRL) (black bar). Each reporter plasmid was transfected three times, and each sample was assayed in triplicate. **(C)** Luciferase reporter assay performed in Hec1a cells co-transfected with the reporter gene containing the SMAD4 3′UTR mutated (pmiRSMAD4 3′UTR-Mut) in the miRs-205, −146a, or −1260b seed sequences (gray bar) alone, the pmiRSMAD4 3′UTR-Mut in each mir seed sequence and miRs-205, −1260b, and −146a (light gray bars), respectively and the negative control (CTRL) (black bar). Bars indicate Firefly Luciferase activity normalized to β-Gal activity ±SD. ^*^p ≤ 0.05 compared to control (CTRL) transfected cells.

Although by different means, Smad4 could be a target of these miRs, one can argue that miR-146a, miR-205, and miR-1260b interact with other unknown targets that down-regulate Smad4 protein levels (18–22) (Figure 4A). To address this concern, we performed a luciferase reporter assay, cloning a 900 bp 3′UTR of human SMAD4 into a pMIR vector (p3′UTRSmad4pMir). Therefore, Hec1a cells were transfected either with miR-146a, miR-205, mir-1260b, or the pre-miR-control, and p3′UTRSmad4pMir vector. As shown in Figure 4B, all miRs decrease luciferase activity of the p3′UTRSmad4pMir compared to control, showing that each miR has a direct effect on their target in this cell lines (Figure 4B). To determine this direct miR-target interaction, we constructed a plasmid with mutagenesis of the three seed sequences (Figure 4C). As expected, we observed only a slight effect on luciferase activity when we compared the wild-type vector with the p3′UTRSmad4pMir mutants in the presence of each miRs overexpressed, showing that the modification of the seed sequence is enough to block the function of each miR (Figure 4C).

### SMAD4 Repression by miRs-205, 146a, and 1260b Induces Proliferation and Migration in Hec1a Cell Lines

Smad4 is involved in the signal transduction pathway of the transforming growth factor ß (TGF-ß) that acts as a tumor suppressor gene in several cancers (26, 27). To gain further insights into how dysregulation of these miRs may play a role in endometrial cancer cells, we performed different assays to study the biological effects of the interaction between these miRs and their target Smad4 into endometrial adenocarcinoma-derived cell lines Hec1a.

**Figure 5:**
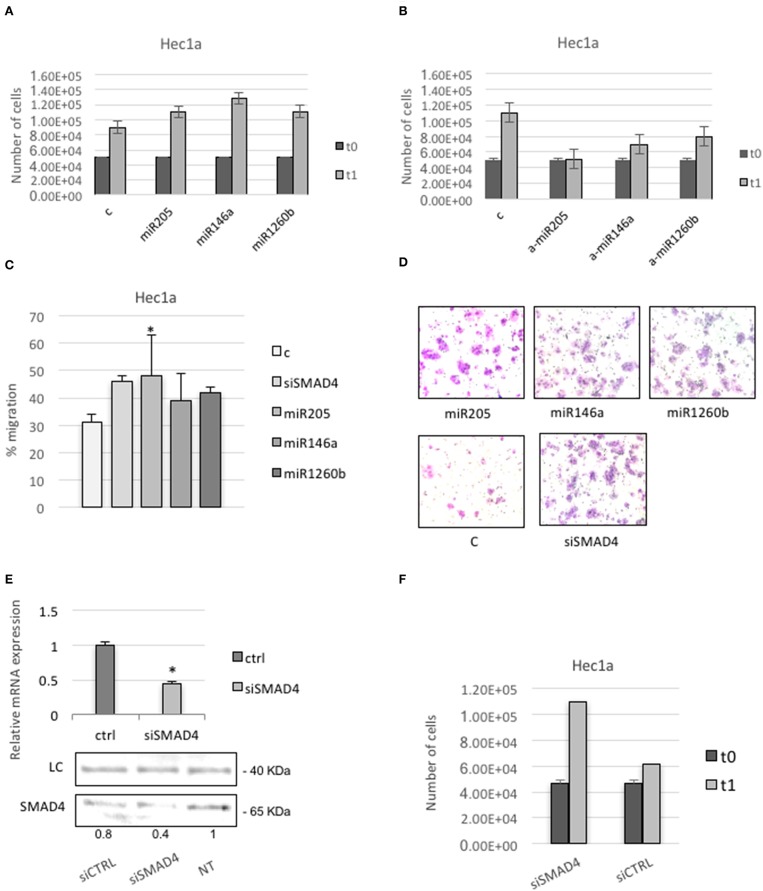
SMAD4 regulation by miRs-205, −146a, and −1260b induces proliferation and migration in Hec1a cells. **(A,B)** Hec1a cells proliferation was measured at 72 h. Cells were transiently transfected with negative control #1 **(C)** and miRs-205, −146a, −1260b in **A** or anti-miRs-205, −146a, and −1260b (a-miRs) in **B**. **(C)** Transwell migration assay shows that overexpression of miRs-205, −146a, −1260b, and siSMAD4 enhance cell migration ability of Hec1a cells. **(D)** Representative photographs of the Transwell migration assay is shown. All data are presented as mean ± SD, ^*^p < 0.05. **(E,F)** Effects of siSMAD4 expression in Hec1a cells. **(E)** RT-PCR (upper panel) and western blot analysis (lower panel) are shown. LC (loading control, β-actin). PCR bars depict SMAD4 expression in control (ctrl) and si transfected cells normalized to β-actin. **(F)** Measure of Hec1a cells proliferation in siSMAD4 and SiCTRL transfected cells is reported. Data represent the mean (from three independent experiments) ±SD. ^*^P < 0.05.

First, we tested cells proliferation. Cells transfected with pre-miRs-146a, −205, or −1260b showed a higher rate of proliferation compared with non-treated or control transfected cells (Figure 5A). Cells transfected with anti-miRs-146a, −205, or −1260b showed a reduction of proliferation compared to control (Figure 5B).

To better understand how the dysregulation of these miRs may change the behavior of endometrial cancer-derived cell line, we examined the influence of Smad4 knockdown on Hec1a cell migration. We found a significant increase in the migration capability in pre-miR-205, −146a, or −1260b transfected cells compared to control, showing that these miRs positively regulate the migration of cultured endometrial cancer cells (Figures 5C,D).

Since miRs may have multiple targets, to ensure that Smad4 mediated the observed effects, we repeated the assay using a specific Smad4 siRNA.

We confirm significant down-regulation of Smad4 through qRT-PCR (Figure 5E). Next, we observed that Hec1a cells transfected with Smad4 siRNA displayed a higher proliferation and migration rate respect to controls (Figures 5C,F).

## Discussion

Identification of molecular markers that can differentiate between AH/EIN and BH are considered to be highly useful for clinical management of patients because hyperplasia with atypical change and/or Endometrial Intraepithelial Neoplasia are associated with a higher risk to progress to cancer (4).

Since there aren't reference markers, the diagnosis is based only on histological features, such as the presence of nucleoli and other atypical characteristics, which are not consistently associated with that diagnosis (9).

Although the new WHO classification is more likely to successfully identify premalignant lesions, the low interobserver reproducibility among gynecological pathologist in diagnosing atypical hyperplasia/EIN should be improved (6, 10, 28–30).

Atypical endometrial hyperplasia/EIN and EC shares several molecular alterations with each other, including microsatellite instability, PAX2 inactivation, mutation of PTEN, KRAS, and CTNNB1 (β-catenin), but there is not a linear accumulation of mutational events leading to cancer (31). Identifying the disease-related miRs will improve the diagnosis and understanding of pathogenesis of these lesions.

Since over 50% of miRs reside in cancer-associated genomic regions, they have been indicated to play an important role as diagnostic biomarkers (13, 32, 33).

Most of the miRs studies on endometrium have been focused on the identification of their implications in EC development, almost neglecting their possible diagnostic role in precursor lesions (34). In fact, several authors showed an altered expression of miRs that may discriminate EC from non-atypical or atypical hyperplasia (9, 14, 21, 35–40).

In particular, expression of five miRs (miRs-182, 183, 200a, 200c, and 205) was significantly higher in EC when compared with complex atypical hyperplasia, simple hyperplasia (SH) and normal endometrial tissue (*P* < 0.05, respectively) (41).

To our knowledge, our study is the first to identify a miRs signature able to discriminate between atypical hyperplasia/EIN and benign endometrial hyperplasia with the capability to better distinguish between low- and high- risk lesions. Identification of miR-target genes and pathways to understand the molecular basis of endometrial cancer pathogenesis is a major challenge, as there are numerous pathways that drive cancer. Accordingly, in this study, we proposed a novel miR-based classification method to categorize the high risk pre-cancerous endometrial lesions.

In fact, we showed a high predictive power, above 90%, using a three miRs-signature (miRs-146a, −205, and −1260b) in distinguishing between non-atypical and atypical hyperplasia/EIN provides a supplementary diagnostic tool when required.

Interestingly, a previous study conducted by Snowdon and colleagues examined a miRs profile in atypical hyperplasia compared to normal proliferative controls. The microarray expression profile shares some important similarities with our data. MiRs-146a, miR-200a, miR-200b, miR-200b-star, and miR-205 resulted up-regulated and miR-542-5p down-regulated in atypical hyperplasia vs. normal endometrium, adding further emphasis to our results (22). The up-regulation of miR-200 family members and miR-205 in EC is observed among different studies, indicating that these miRs may play a role in driving oncogenesis in the endometrium (21, 35, 37, 42, 43).

Some authors showed that miR-205 is a negative prognostic marker for EC and its levels were significantly increased in endometrial cancer cell lines and endometrial tumors compared to normal tissues (25, 26). Mir-205 is directly involved in PTEN regulation that represents one of the most commonly investigated markers implicated in endometrial tumorigenesis (44). Our results have enforced this effect indicating a remarkable influence of miR-205 on regulating essential target genes involved in different signal pathways in endometrial cells. On the other hand, Lacey et al. have shown that a loss of expression of PTEN status was not associated with progression risk of endometrial hyperplasia (45).

Even if EC seems to be characterized by elevated expression of miR-205, a recent study conducted by Wilczynski showed that higher levels of miR-205 may be a marker of an early stage disease and is associated with a more favorable prognosis, whereas patients with lower levels of miR-205 had worse survival (42).

No evidence has revealed the dysregulation of miRs-1260b and −146a in EC, suggesting that up-regulation of these miRs may be specific of AH/EIN.

Interestingly, authors found that a single nucleotide polymorphism (SNP) rs2910164 G>C within miR-146a is associated with the increased risk of gastric cancer and papillary thyroid carcinoma (46, 47). A recent publication showed that overexpression of miR-146a inhibited cell proliferation, enhanced apoptosis, and increased sensitivity to chemotherapy drugs in epithelial ovarian cancers cells showing, therefore, that the role of miR-146a is still to be elucidated (48).

MiR-1260b has been found to be highly expressed in the prostate, renal cell, and in colorectal carcinomas (49, 52). Recently, it was demonstrated that in Hepatocellular carcinoma, MiR-1260b promotes cell migration and invasion through the G-protein signaling 22 (50).

All of this evidence confirmed that our identified miRs regulate genes involved in different signal pathways that may trigger the endometrial cellular transformation.

Thus, we investigated a possible common pathway that could be regulated by these miRs and could be implicated in cellular transformation, and we found that they target SMAD4. Also noteworthy are several other reports, which demonstrated a direct interaction of these miRs and this transcript (51–53).

Smad4 is a gene implicated in several cancers, including EC, albeit its role in endometrial carcinogenesis is yet not clear (54–56).

Impairment of the Smad pathway results in escape from growth inhibition and leads to the promotion of cell proliferation, contributing to carcinogenesis (57).

The disturbances in Smad proteins expression and/or differences in their intracellular distribution, that trigger a TGF-β signaling pathway deregulation, it was reported in endometrial carcinomas, but it is still not well-understood (58). The region within 18q21 where Smad4 is located is frequently deleted in endometrial carcinomas, showing its involvement in EC, however, an immunohistochemical study showed that inactivation of this gene occurs infrequently in this tumor.

Changes in the expression of the TGF-β signaling cascade in type I ECs seem to be associated mainly with deregulation of TGF-β receptors and SMAD expression at the protein level, indicating SMAD4 as a central molecule of this pathway (59, 60).

Thus, the potential pathogenic role of SMAD4 in endometrial hyperplasia is supported by our finding, albeit further studies are required to understand its biological and diagnostic role in this environment.

Our results clearly demonstrated that overexpression of miRs-146a, −205, and −1260b induced Hec1a proliferation and migration through SMAD4 inhibition, providing an insight into the possible mechanisms underlying the function of these miRs in endometrial hyperplasia.

Thus, our work highlights the relevance of miRs in regulating cellular processes that may ultimately lead to tumorigenesis.

Taken together these results strongly show that miRs-205, −146a, and −1260b contribute to enhancing proliferation and migration properties of endometrial cancer cells through Smad4 inhibition.

In conclusion, distinguishing between hyperplasia and true pre-cancerous lesions has significant clinical implications because distinct endometrial pre-cancerous conditions require intervention. Thus, we proposed a three miR-signature (146a −205, −1260b) as a potential biomarker for diagnosis of atypical endometrial hyperplasia/EIN that could have a significant impact on treatment decisions. Furthermore, the regulatory capability of these three miRs on cell proliferation and migration, possibly through impairment of TGF-β signaling Smad4-mediated, highlights their crucial role in endometrial hyperplasia outcome.

Although we believe that this study represents a step forward in investigating the molecular relationship between miR deregulation and EIN lesions, we analyzed a relatively small group of patients, and therefore, a prospective analysis is needed to strengthen the accuracy of our results.

## Ethics Statement

This study was carried out in accordance with the recommendations of name of guidelines, name of committee with written informed consent from all subjects. All subjects gave written informed consent in accordance with the Declaration of Helsinki. The protocol was approved by the IRB committee.

## Author Contributions

SG and AV: study conception and design and drafting the manuscript. SG, VA, RC, OF, CD, and MP: methodology. SG, SV, and AV: analysis and interpretation of data. DC, AP, FF, and AV: access to clinical data. SG, DC, AP, FF, and AV: critical revision.

### Conflict of Interest Statement

The authors declare that the research was conducted in the absence of any commercial or financial relationships that could be construed as a potential conflict of interest.
